# Anti‐microtubular activity of total alkaloids and aqueous extract of *Detarium microcarpum* a medicinal plant harvested in Mali

**DOI:** 10.1007/s00709-024-02003-3

**Published:** 2024-11-07

**Authors:** Niaboula Dembele, Aimé Ainin Somboro, Nah Traore, Mamadou Badiaga, Salimatou Cisse, Mody Cisse, Peter Nick

**Affiliations:** 1 Institute of Applied Sciences, USTTB, Bamako, Mali; 2 Faculty of Sciences and Techniques, USTTB, Bamako, Mali; 3 Faculty of Pharmacy, USTTB, Bamako, Mali; 4 Present Address: Karlsruhe Institute for Technologies, KIT, Karlsruhe, Germany

**Keywords:** Anti‐microtubule activity, Mortality assay, *Detarium microcarpum*, Alkaloids, Aqueous extract, Spinning disc microscopy, Sikasso, Sanankoroba, Mali, Traditional African medicine

## Abstract

*Detarium microcarpum*, is a species confined to drier regions of west and central Africa used to treat various diseases including cancer. Phytochemical screening revealed the presence of secondary metabolites (alkaloids) The aim of this work is to study the effect of total aqueous extracts and alkaloid fractions from *D. microcarpum* leaves, bark and roots on *Nicotiana tabacum* L. cv. ‘Bright Yellow 2’ (BY-2) tobacco cell line GFP-TuA3 expressing a *N*-terminal fusion of GFP. The plant was harvested in two different regions of Mali with a contrasting climate. The effects of the extracts on the microtubules was followed by spinning disc confocal microscopy. We showed that the anti-microtubular effect of the extracts is dose-dependent, depends of the sampling site and the part of the plant used. Total alkaloids extracted of *D. microcarpum* bark have more effect on microtubules than leaf and root. The bioactivity‐guided fractionation should be used to screen out the biologically active compounds of the total alkaloid extracts of the bark of *D. microcarpum*.

## Introduction

The use of plants for healing has accompanied humans from the very beginning (for review see Gurib-Fakim 2006). Even wild chimpanzees have been observed to use specific plants for medicinal purpose, suggesting that the knowledge on the curing power of plants predates humanity (Page et al. 1992). The search for the compounds responsible for the medicinal activity of such plants has often led to the discovery of new drugs (for reviews see Shen 2015 and Bernardini et al. 2018). Interestingly, many of these plants are toxic and it was often this toxicity, which helped to identify the active molecule. The economic impact of such plant-derived drugs can be enormous, as illustrated for the alkaloids Paclitaxel (from the Pacific Yew, *Taxus brevifolia*), Morphine (from the Opium Poppy, *Papaver somniferum*) or Artemisinin, a sesquiterpene from the Sweet Wormwort (*Artemisia annua*), so far, the most efficient ailment against Malaria.

Especially in developing countries, plant-based healing has remained the predominant therapeutical approach. For instance, due to low income and often long distances from urban treatment centres, many people in Africa commonly use medicinal plants for cancer treatment (Goabaone et al. 2023). Causing nearly 10 million deaths in 2020, cancer is one of the leading causes of death worldwide (WHO 2020). Access to treatments such as surgery, radiotherapy and chemotherapy is highly unequal, though. Whilst in wealthy, industrialised countries more than 90% of patients can profit from these treatments, in poor countries less than 15% of patients get the chance for such treatments according to the latest numbers of the WHO (2020). This inequality of access to modern therapy is reflected by a strong inequality in survival. Around 70% of deaths from cancer occur in low and middle-income countries many of which are located in Africa (Goabaone et al. 2023). In the majority of sub-Saharan African countries, health services suffer from severe limitations in the resources needed for early detection, diagnosis, treatment and adequate care of cancer. In these countries, plant-based traditional medicine is deeply rooted in the culture and in many societies also embedded in a traditional system of magic-religious beliefs and practices that has been passed on from immemorial time (Guedje et al. 2013). To use plants as source of medicinally active compounds for cancer treatment is not limited to sub-Saharan ethnomedicine, though around 70% of compounds used in chemotherapy of cancers, including Paclitaxel or the Vinca alkaloids, have been identified in medicinal plants (for review see Newman et al. 2003). In general, alkaloids seem to have large potential as anti-cancer compounds (for review see Mondal et al. 2019).

For the dry regions in West and Central Africa, the tree *Detarium microcarpum* shows potential as source for anti-tumour compounds. This tree is confined to the dry regions of West and Central Africa. Due to its extensive, horizontal root system, it cannot only grow in forests, but also cope with the more arid conditions of savannahs. Ethnopharmacological investigations amongst traditional healers shows that this tree is widely used, often in region-specific therapeutic applications (Dembele et al. 2022). The sweet fruits, but also other parts of this tree, are widely used to cure diseases as different as meningitis, tuberculosis, haemorrhoids, diabetes, malaria or inflammations (a comprehensive review is given by Dogara 2022). This plethora of applications also includes the therapeutic use against cancers. In fact, extracts from *D. microcarpum* have been reported to arrest the cell cycle in breast cancer cell lines, and the resulting mitotic catastrophe leads to the apoptotic breakdown of these cells (Mohammed et al. 2016; Adebayo et al. 2019).

Important anti-cancer drugs of plant origin, such as Paclitaxel, Vincristine or Parthenolide, exert their therapeutic effect through interaction with microtubules (for review see Altmann and Gertsch 2007). There are several modes of action – in many cases, such as for vincristine, the compounds sequester αβ tubulin heterodimers from integration into the microtubules, such that the microtubule will disappear based on its innate turnover (Owellen et al. 1976), a few compounds, such as Paclitaxel, block the depolymerisation of microtubules by binding to □-tubulin (Parness and Horwitz 1981), and a third group of compounds, such as Parthenolide are altering the post-translational modifications and, thus, the interaction with associated proteins that modify tubulin dynamics and organisation (Fonrose et al. 2007). Although the term ‘microtubule-disruption’ is widely used in the literature, we will refrain from this terminology, because a true disruption of pre-existing microtubules seems to be rare, if it exists at all (Mizuno and Suzaki 1991). We will in the following remain on the phenomenological level and instead use the term microtubule eliminating to describe such activities.

Since microtubules are common targets for anti-tumour compounds, it is straightforward to ask, whether also extracts from *D. microcarpum* harbour anti-microtubular activity, and if so, whether this activity can be associated with specific components of the microtubular cytoskeleton. The behaviour of microtubules does not only depend on the building block, heterodimers of □- and □-tubulin, but is also regulated by multiple associated proteins that facilitate or inhibit assembly, promote disassembly, cross-connect microtubules or link them to other proteins or organelles. These so-called microtubule-associated proteins (MAPs) have been subject to pronounced evolutionary diversification, which is linked with plant-specific microtubule arrays such as cortical microtubules, preprophase band or phragmoplast that are not found in animal cells and convey functions linked with the peculiar requirements of the walled plant cells that grow by uptake of water and have to separate their daughter cells by a centrifugally extending cell plate (for review see Nick 2008). In contrast, the tubulins, along with a limited set of MAPs involved in microtubule nucleation, display a high degree of conservation. It is possible, for instance, to follow the dynamic reorientation of plant microtubules by microinjection of tubulin that had been purified from porcine brain and conjugated to fluorescent dyes. The foreign tubulins are readily integrated and functionally fully equivalent to the native tubulins of the plant cell (Himmelspach et al. 1999). A possible strategy to find out, whether a compound with a potential anti-microtubular activity is acting on the tubulin itself, is a test on plant cells, where the majority of associated proteins differs from those in mammalian cells. If an anti-microtubular activity is observed in plant cells, it is probably due to interaction with tubulin and, thus, also to be expected in mammalian cells. Following this rationale, we used, in the current study, a tobacco BY-2 cell line, where microtubules were tagged with the Green Fluorescent Protein (GFP) and tested, whether total extracts from different parts of *D. microcarpum* collected at two different sites in Mali, exhibit anti-microtubular activity. In fact, we can show a dose-dependent and time-dependent elimination of microtubules in response to such extracts, and we can show that this activity is potentiated in the alkaloid fraction. Our bioassay shows further that the anti-microtubular activity depends on donor tissue (leaf, bark or root) and also on collection site. Similar to the breast cancer cell line, where extracts from *D. microcarpum* elicit apoptotic cell death (Adebayo et al. 2019), we also observe that the elimination of microtubules is later followed by cell death, corroborating the existence of mitotic catastrophe also for plant cells.

## Material and methods

### Plant material and sampling

Leaves, bark and total roots of *Detarium microcarpum* Guill. & Perr. were collected from Tabarako (region Sikasso) and Bougoula (region Sanankoroba), between October to November 2018. These sites were selected for the following criteria: (1) Use of *D. microcarpum* in the local medicinal practice. (2) Contrasting climatic conditions between the sites. Sikasso is characterised by sufficient rainfall during the raining season with a vegetation ranging from wooded savannahs to gallery forests. In contrast, in Sanankoroba, rains are falling irregularly. (3) Abundance of *D. microcarpum*, and (4) low level of environmental pollution. The material was authenticated by the Department of Traditional Medicine (DMT) based on morphological traits of the leaves and vouchers are kept at the National Institute of Public Health (INSP) in Bamako (Mali) under the registration number 3053/DMT.

### Preparation of total aqueous extract

Aqueous total extracts were generated as described in Dembele et al. (2023). In brief, 20 g of powdered plant material were boiled in 200 mL of distilled water under reflux whilst stirring for 15 min. The resulting solution was then allowed to cool down and filtered under vacuum. The filtrate was then evaporated to dryness in the oven for 24 h. The solid, dry residue was then kept at 4 °C until further analysis of the biological activity. Prior to the experiment, the dry residue was dissolved in H_2_O at 90 °C to a stock concentration of 0.5 mg^.^mL^−1^.

### Preparation of total alkaloid fractions

**Fig. 1:**
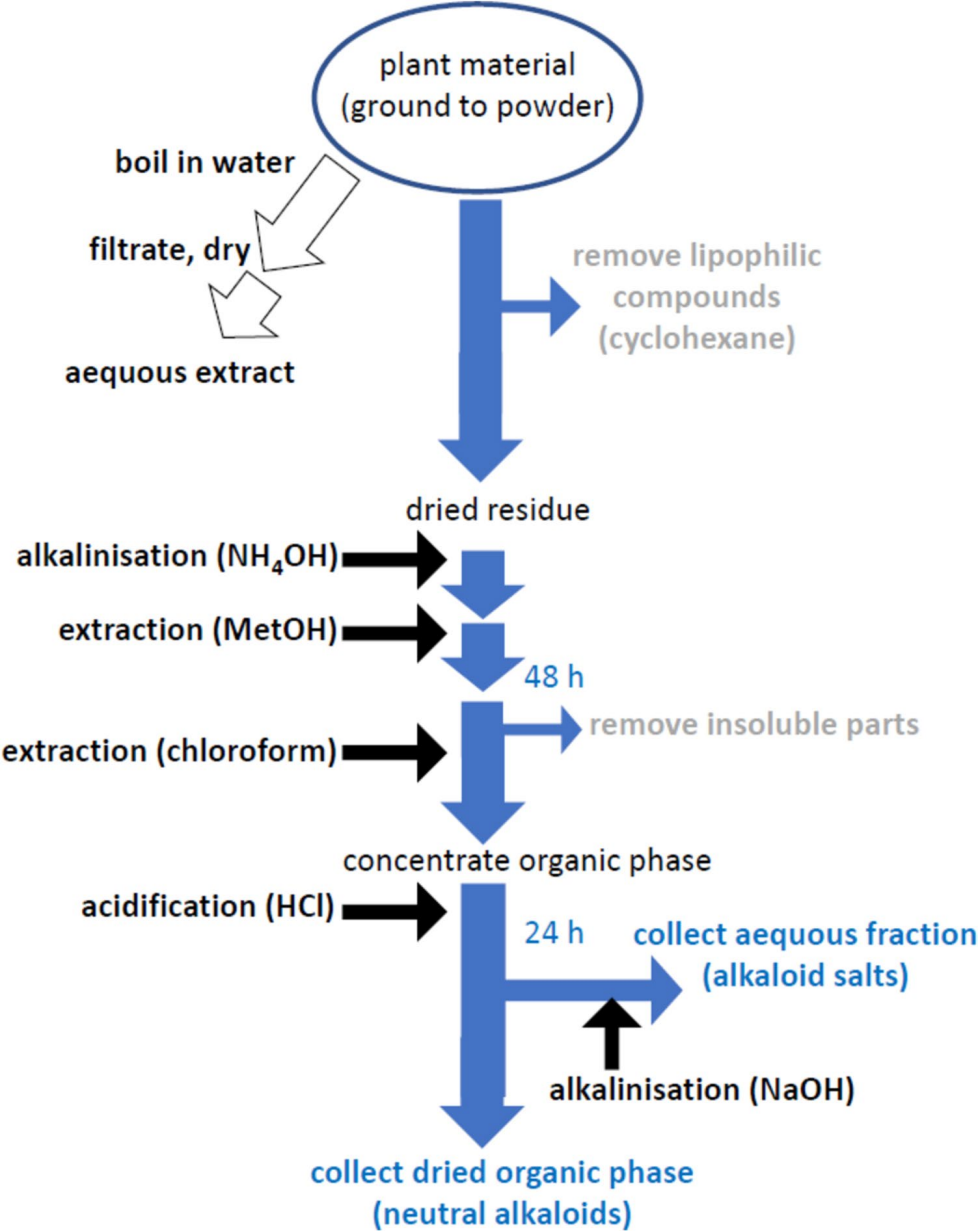
Generation of aqueous extract and alkaloid fractions from *Detarium microcarpum* leaves, bark and roots

*Detarium microcarpum* alkaloids were extracted from bark, root and leaves using material that had been thoroughly dried at ambient temperature using a standard protocol (Fig. 1) making use of the chemical properties of alkaloids to act as proton acceptors under neutral or acidic conditions, whilst being relatively hydrophobic, due to their organic nature (for review see Klein-Júnior et al. 2016). The plant material was ground to a powder and then macerated in cyclohexane as an apolar solvent for 24 h to remove lipophilic compounds such as waxes, terpenes or pigments that might disturb extraction by forming emulsions. The supernatant was removed by vacuum filtration, and the residue dried. Prior to extraction, the material was moistened with ammonium hydroxide (35% w/v, 100 mL per 100 g of plant material) to obtain alkaline conditions before methanol (in case of leaves 300 mL, in case of bark and root 400 mL per100 g of plant material) was added to extract the alkaloids. The mixture was then stirred for 48 h at ambient temperature, and filtered again by vacuum filtration, before adding chloroform (around 1 L per 100 g of plant material). The chlorinated organic phase was then concentrated under reduced pressure with a rota-vapour. The concentrate was then extracted with 5% of hydrochloric acid (150 mL per 100 g of plant material) for acidification, and the mixture stirred subsequently for 24 h. This acidic aqueous phase containing alkaloid salts was then recovered using a separatory funnel and made alkaline with NaOH (1 M). The chloroform phase was then dried under vacuum and the residual harbouring the neutral alkaloids was collected.

## Cultivation and treatment of tobacco cells

The study was conducted with suspension cells of *Nicotiana tabacum* L. cv. ‘Bright Yellow 2’ (BY-2) tobacco cell line GFP-TuA3 expressing a N-terminal fusion of GFP to tobacco tubulin □3 under control of the CaMV 35S promoter (Kumagai et al. 2001) in a modified Murashige-Skoog medium, supplemented with 200 mg^.^L^−1^ of KH_2_PO_4_, 100 mg^.^L^−1^ of *myo*-inositol, 1 mg^.^L^−1^ of thiamine and 0.9 µM of the artificial auxin 2,4-dichlorophenoxyacetic acid, pH 5.8 inoculating 1 g of fresh weight into 30 ml of medium at weekly intervals on an orbital shaker (IKA Labortechnik, http://www.ika.de) at 150 rpm at 26 °C in the dark. To assess the effect on microtubular integrity, aqueous extract from leaves, bark or roots of *Detarium microcarpum* was applied at a concentration of 0.5 mg^.^mL^−1^ into 2 mL of cell suspension and a treatment time of 2 h at 26 °C in Petri dishes of 35 mm diameter, 2 mL microtiter wells on an orbital shaker at 150 rpm. Cells were used for the experiment at the peak of their mitotic activity, at day 3 after sub-cultivation. As positive control, the antimicrotubular herbicide oryzalin was used at 10 µM. In some experiments, a dose–response curve was recorded for aqueous bark extract of *D. microcarpum* from Sikasso using a treatment time of 2 h. For a time-course experiment, a higher concentration (5 mg^.^mL^−1^), again for bark extract from Sikasso, was used. To probe for the effect of alkaloids, 0.05 mg^.^mL^−1^ or with 0.50 mg^.^mL^−1^ of the neutral alkaloid fraction generated from leaf, bark or root sampled in Sanankoroba was used. Due to limitations in volume, the incubation took place in 1.5-mL reaction tubes in volumes of 500 µL suspension under continuous shaking (150 rpm) at 26 °C in the dark. Aliquots of the treated cells were used for microscopy. Since extracts and alkaloids were dissolved in DMSO, a solvent control of 0.5% DMSO, corresponding to the highest concentration in the assay, was included as well as 10 µM oryzalin (Sigma-Aldrich, Deisenhofen) as positive control for microtubule disintegration.

### Monitoring and quantifying microtubule integrity

The effect of aqueous extracts and alkaloid fractions on microtubules could be monitored by virtue of the green fluorescence of tubulin. The response of microtubules was followed by spinning disc confocal microscopy (Yokogawa CSU-X1 5000) using an AxioObserver Z1 and a 63 × LCI‐NeofluarImmCorr DIC objective (NA 1.3), exciting fluorescence with an Ar-Kr laser (488 nm emission). The confocal stacks were recorded and processed with the ZEN Blue software (Zeiss, Jena). Microtubule integrity was quantified as described in Guan et al. (2021). The principle of this quantification are intensity profiles collected parallel to the elongation axis using the freeware ImageJ (https://imagej.nih.gov/ij). Microtubules will appear as peaks in the profile, whilst soluble tubulin dimers will appear as troughs between the peaks. With progressive disintegration, the number and height of peaks over the troughs will decrease. This can be quantified by determining the first derivative of intensity, yielding a positive value in the rising, and a negative value in the trailing flank of an intersected microtubule. Random signals deriving from noise in the photomultiplier are filtered out, by adding subsequent values in the first derivatives. With progressive disintegration the steepness of the peaks and the depth of the troughs will progressively equalise, which can be scored by determining the standard deviation of pixel intensity. Although all images were recorded at the same exposure time, potential differences in laser settings are normalised by dividing this standard deviation by the maximal intensity of the profile. Data represent means and standard errors from 20 to 30 individual cells per data point for the experiments with extracts, whilst for the experiments with alkaloids, a lower number of 10–19 individual cells were scored.

### Mortality assay

The cytotoxic activity of the alkaloid fraction generated from the different organs of *D. microcarpum* collected at the two sites was probed in the same tobacco cell strain used for monitoring the microtubule response, i.e., *Nicotiana tabacum* L. cv. ‘Bright Yellow 2’ (BY-2) tobacco cell line GFP-TuA3 expressing a N-terminal fusion of GFP to tobacco tubulin □3 under control of the CaMV 35S promoter (Kumagai et al. 2001). The alkaloids were diluted from a stock solution of 10 mg/mL in dimethyl sulfoxide (DMSO) to working concentrations of 25, 50 and 100 µg/mL) to 2 mL of cell suspension in Petri dishes of 35 mm diameter. The cells were used at the peak of mitotic activity, at day 3 after sub-cultivation, scoring the resulting mortality 72 h after addition of the alkaloids. During this period, the cells were cultivated under the same conditions as usual, shaking with 150 rpm on an orbital shaker, in the dark, at 26 °C. As solvent control we used 0.5% DMSO (corresponding to the intermediate solvent concentration in the assays) was prepared, in parallel with another negative control, consisting of cell medium only. Mortality was scored using the Evans Blue Dye Exclusion Assay (Gaff and Okong’o-Ogola 1971). In brief, 800 µL of of 2.5% (w/v) Evans Blue (Sigma-Aldrich, Deisenhofen) were added to each sample, consisting of 200 µL of cell suspension. Unbound dye was washed out with three times of double distilled water for 5 min per passage, using a custom-built chamber (Nick et al. 2000). Living cells, due to the integrity of their membrane, exclude the dye and remain white, whilst dead cells are stained blue. Data represent mean and standard errors from three independent experimental series, each measurement representing 500 individual cells.

### Molecular phylogeny

To assess the identity and phylogenetic relationship of the two samples of *D. microcarpum* collected in Sikasso and Sanankoroba, we used genetic barcoding, using the plastidic markers RbcL and the trnH-psbA intergenic spacer. These markers have wide coverage in public databases combining good discriminative resolution with good sequencability and have therefore been integrated into the set of barcodes for land plants (CBOL 2009). Bark material was shock-frozen in liquid nitrogen and homogenised by a TissueLyser (Qiagen, Hilden, Germany). Genomic DNA was extracted with cetyl trimethyl ammonium bromide (CTAB) according to Doyle and Doyle (1987). After incubation in 1 mL of 1.5% w/v CTAB for 1 h at 65 °C, the samples were mixed with 630 µl of chloroform/isoamylalcohol (24:1), shaken horizontally for 15 min, and spun down for 10 min at 4 °C with 17,000 g. The upper aqueous phase with the DNA, was precipitated with 66% of ice-cold isopropanol, and the DNA sedimented by centrifugation (10 min, 17,000 g, 4 °C). The sediment was washed with 1 mL 70% EtOH, and the EtOH removed by drying in a vacuum centrifuge for 15 min, and the DNA precipitate finally dissolved in 50 µL nuclease-free water (Lonza, Biozym) containing 5 µg RNAse A (Qiagen, Hilden, Germany). Concentration and purity of the eluted DNA were spectrophotometrically determined (NanoDrop ND-100, peqlab). The different barcoding markers (*rbcL*, *trnH-psbA igs*) were amplified from 75 ng template DNA in a reaction volume of 30 µL with 3 µL 10 × reaction buffer (Thermopol, New England Biolabs), 3 µL of Bovine Serum Albumine (10 mg.mL^−1^), 0.6 µL of dNTPs (200 µM, New England Biolabas), 0.6 µL of both oligonucleotide primers (Merck, Darmstadt) and 0.3 µL of Taq polymerase (5 U, New England Biolabs). Amplification was conducted with an initial denaturation step of 120 s at 95 °C followed by 35 cycles of denaturation at 95 °C for 30 s (45 s in case of *trnH-psbA igs*), annealing at 60° for 30 s, synthesis at 68° for 60 s, and a final elongation at 68° for 300 s. Amplicons were evaluated by gel electrophoresis using NEEO ultra-quality agarose (Carl Roth, Karlsruhe, Germany). DNA was visualised using Midori Green (NIPPON Genetics EUROPE, Germany) under blue light excitation. The fragment sizes of the amplicons were determined by a 100 bp size standard (New England Biolabs). The amplified DNA was purified using the protocol of the MSB Spin PCRapace Kit (Stratec), and the amplicons were read in both directions by Sanger sequencing (Eurofins, Konstanz, Germany). The two reads were aligned after reverse complementing the reverse read using the MUSCLE algorithm of the software package MEGA7 (https://www.megasoftware.net/). Details of the primers and the GenBank accession numbers for the barcodes are given in Table 1. For phylogenetic analysis, the sequences obtained from the two *D. microcarpum* samples were used as baits for a BLAST search in GenBank to the NCBI browser. Sequences from related taxa and appropriate outgroups were collected by means of the Taxonomy View routine. Phylogenetic trees were inferred from the respective alignments using the Neighbour-Joining algorithm and were visualised using the Tree Explorer integrated into MEGA. Bootstrap values were determined using 1000 replications.

**Table 1:** Oligonucleotide primers used in the current study. Amplicon lengths are predicted for *Detarium* taxa investigated in the current study

Target	Sequence	Amplicon (bp)
RbcL	F: 5-ATGTCACCACAAACAGAGACTAAAGC-3 R: 5-CGTGGTGGACTTGATTTTAC-3	599
matK	F: 5-ACCCAGTCCATCTGGAAATCTTGGTTC-3 R: 5-CGTACAGTACTTTTGTGTTTACGAG-	878
trnL-F igs	F: 5-CGAAATCGGTAGACGCTACG-3 R: 5-ATTTGAACTGGTGACAGAG-3	803–1209
trnH-psbA igs	F: 5-GTTATGCATGAACGTAATGCTC-3 R: 5-CGCGCATGGTGGATTCACAATCC-3	420–446

## Results

### Aqueous extracts from *Detarium microcarpum* eliminate microtubules

To emulate the ethnopharmaceutical practice, we first studied aqueous extracts, moreover, since the amount of the alkaloid fractions was limited. To screen for potential anti-microtubular activities of aequous extracts from *Detarium microcarpum*, we used the microtubule marker line *Nicotiana tabacum* cv. Bright Yellow 2 GFP-TuA3, where tobacco tubulin □3 was expressed as fusion with GFP to the N-terminus under control of the constitutive CaMV 35S promoter (Kumagai et al. 2001).

**Fig. 2:**
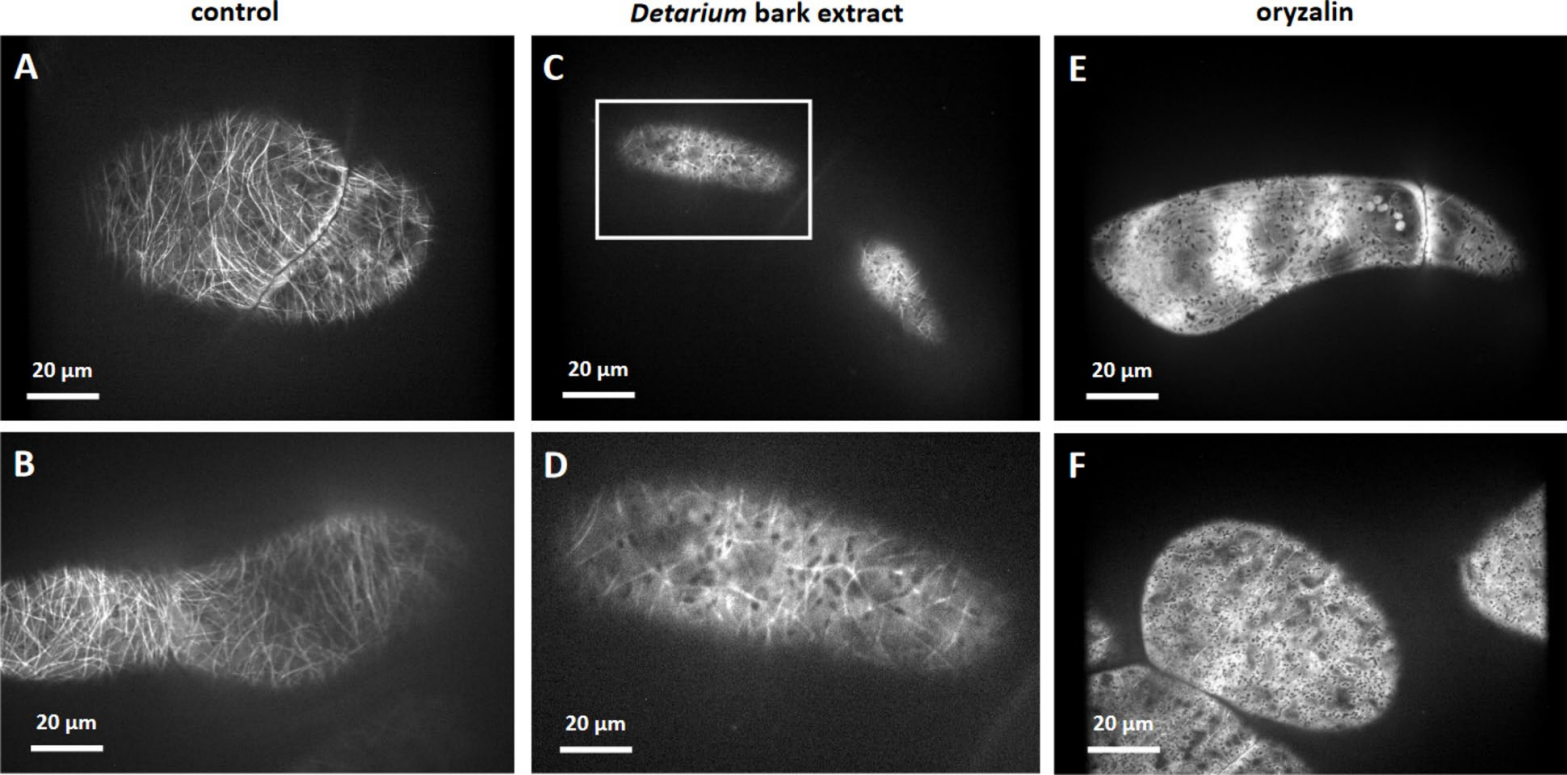
Representative cells of the tobacco BY-2 cell line GFP-TuA3 expressing a *N*-terminal fusion of GFP to tobacco tubulin □□ under control of the CaMV 35S promoter to visualise microtubules *in vivo*. Treatment for 2 h either with culture medium alone (**A**, **B**), an aqueous bark extract of *Detarium microcarpum* (collection site Sanankoroba) at 0.5 mg^.^mL.^−1^ (**C**, **D**) or the microtubule eliminating herbicide oryzalin at 10 µM (**E**, **F**). Insert in **C** is zoomed in **D** to show the partial disassembly of microtubules and the high background of solublte GFP-tagged tubulin. Confocal sections in the cortical region obtained by spinning disc microscopy are shown. Since these are single confocal sections, the illuminated area differs epending on the shape of the cell (round versus flat in diameter)

Cells were challenged by 0.5 mg^.^mL^−1^ of an aqueous bark extract of *D. microcarpum* collected at Sanankoroba, and the potential response allowed to develop for 2 h at 26 °C. To ensure non-invasive conditions, the cell suspension remained on the shaker. A negative control, where cells were kept in cultivation medium without extract confirmed that the conditions as such did not perturb microtubules. Confocal sections of the region right under the plasma membrane (Fig. 2A, B) revealed arrays of numerous and mostly parallel bundles of cortical microtubules, whilst the surrounding cytoplasm showed only a weak fluorescence indicating that the pool of disassembled tubulin heterodimers was low. Treatment with the aqueous bark extract of *D. microcarpum* produced a clearly different result (Fig. 2C, D). Here, only a few microtubules remained, and these appeared disordered. This was accompanied by a high background of soluble GFP-tagged tubulin. Additionally, black spots, embedded into this fluorescent background, became observable. Size and shape of these black spots suggest that these are negative images of plastids surrounded by the fluorescent cytoplasm. To assess, how a complete response would look like, we included a positive control with 10 µM of oryzalin, a herbicide that eliminates microtubules by sequestering the heterodimers, such that the microtubule disappears from its innate turnover. As to be expected, this treatment eliminated microtubules completely (Fig. 2E, F), whilst cells exhibited a strong soluble cytoplasmic fluorescence, which, again, was excluded from ovoid spots corresponding to the *bona-fide* plastids.

### The anti-microtubular effect of the aqueous extract is dose-dependent

However, to compare efficacy, we used the concentration determined from the bark as effective. To test, whether the anti-microtubular effect of the *D. microcarpum* bark extract was specific, we constructed the dose–response relation, scoring microtubule integrity based on quantitative image analysis using a strategy that had been successfully employed for bio-activity guided fractionation of anti-microtubular activities in the TCM plant Northern Ban Lan (Guan et al. 2021). In brief, intensity profiles were collected parallel to the long axis of the cell. In those profiles, microtubules will appear as peaks, separated by troughs representing the intervening cytoplasm. When microtubules disintegrate, the troughs will be filled due to the signal from soluble GFP-tubulin. Instead, the number and height of the microtubule ridges will drop. This change can be picked up by scoring the standard deviation of the first derivative in the intensity profile. Thus, an intact microtubule array will yield a high value, which will progressively dissipate, when microtubules disintegrate and fill the space between them with soluble tubulin dimers.

**Fig. 3:**
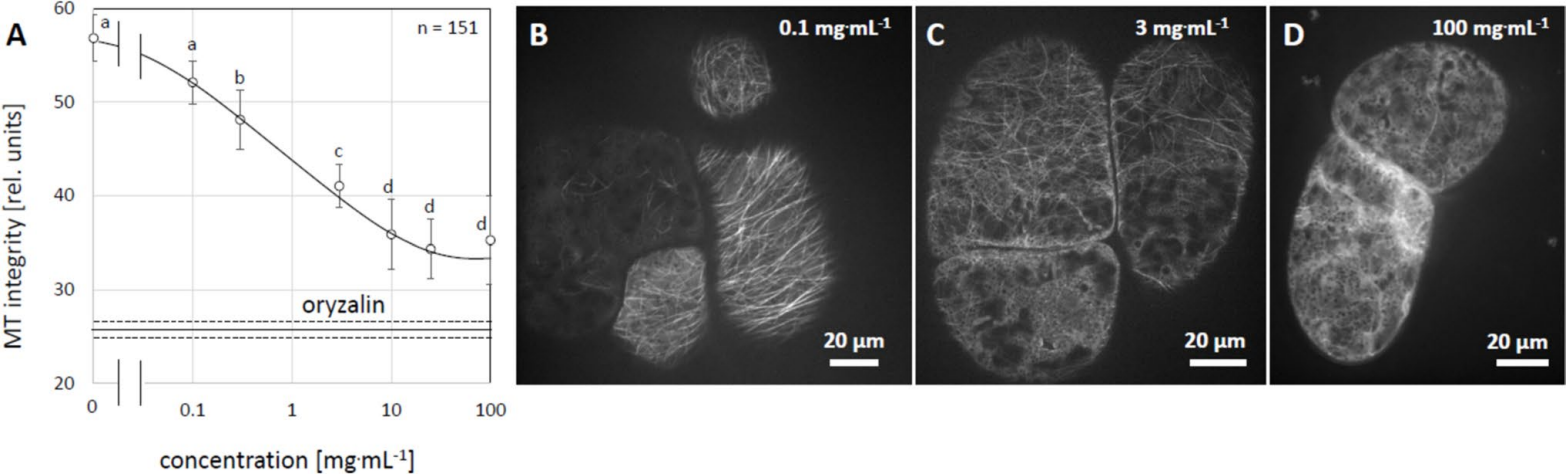
**a** Dose–response relation for microtubule disintegration by an aqueous extract from the bark of *D. microcarpum* (collection site Sanankoroba) in GFP-TuA3 cells. Treatment time was 2 h, 10 µM of oryzalin was used as positive control (solid line, dotted lines show standard errors). Different letters show significant differences between different genotypes and treatments according to a Duncan test at *P* < 0.05). **b-d** representative cells after treatment with either 0.1 mg^.^mL^−1^ (**b**), 3 mg^.^mL^−1^ (**c**) or 100 mg^.^mL^−1^ (**d**), respectively

Again, using an incubation time of 2 h, we observed that microtubule integrity deteriorated progressively, when the concentration of the extract was raised (Fig. 3b–d). Upon quantification, microtubules of untreated control cells produced an integrity score of almost 60, whilst complete elimination of microtubules by oryzalin produced a score of 25–30. The cells treated by the aqueous *D. microcarpum* extract produced scores that dropped progressively with increasing concentration (Fig. 3a). From 0.3 mg^.^mL^−1^, the values became significantly lower compared to the control. From 10 mg^.^mL^−1^, saturation was reached at values of 30–35, which was almost reaching the levels seen for oryzalin. Due to constraints in material for leaf and root extracts, it was not possible to conduct a dose–response curve for those tissues.

### The anti-microtubular effect depends on source tissue and sampling site

**Fig. 4:**
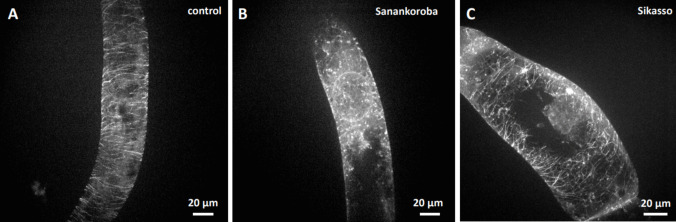
Effect of aqueous extracts (0.5 mg. mL^−1^) from bark of *D. microcarpum* collected at two different sites in Mali in GFP-TuA3 cells Treatment time was 2 h. **a** mock control. **b** extract from bark collected in Sanankoroba. **C** extract from bark collected in Sikasso. Geometrical projections from confocal z-stacks are shown

To test, how the anti-microtubular activity of the extracts related to the different source tissues coming from different sampling sites, we compared the effect using incubation for a duration of 2 h at a fixed concentration (50 mg. mL^−1^), a concentration that in case of bark collected at Sanankoroba was close to maximal inhibition (Fig. 3a). Indeed, we found that the bio-activity of the extracts differed significantly. For instance, bark extract from Sanankoroba eliminated microtubules more thoroughly (Fig. 4b) compared to bark extract from Sikasso (Fig. 4c), where still some microtubules were still visible despite a noteworthy disruption.

**Fig. 5:**
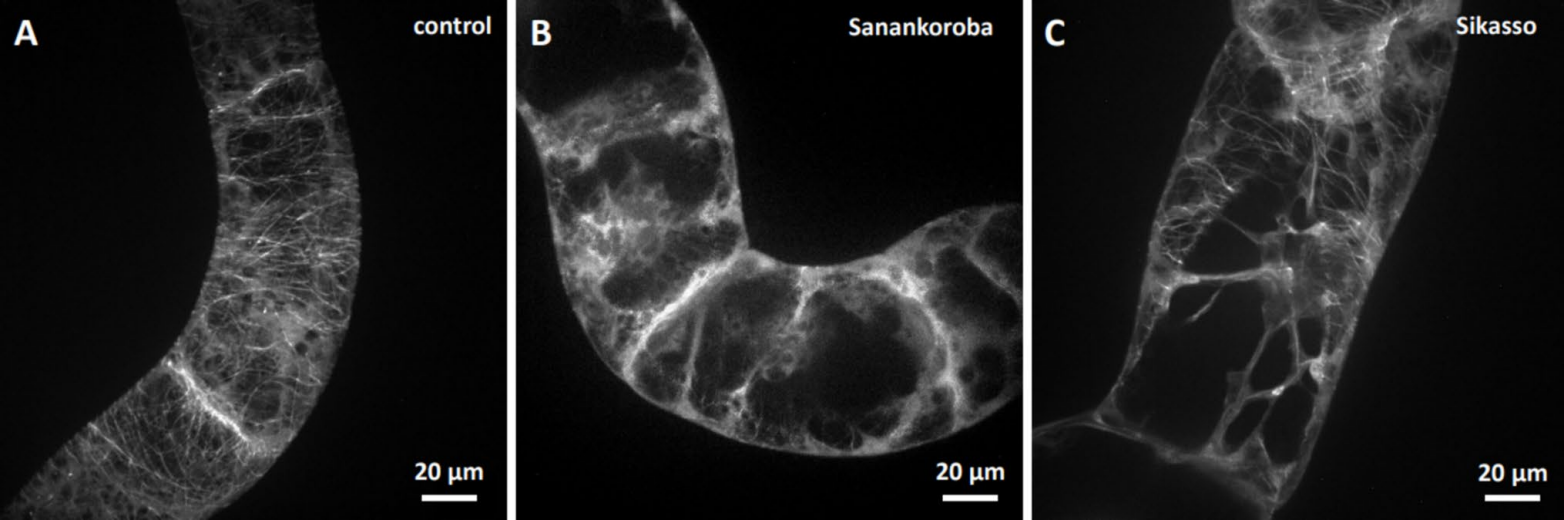
Effect of aqueous extracts (0.5 mg. mL^−1^) from leaves of *D. microcarpum* collected at two different sites in Mali in GFP-TuA3 cells. Treatment time was 2 h **a** mock control **b** extract from leaves collected in Sanankoroba **c** extract from leaves collected in Sikasso. Geometrical projections from confocal z-stacks are shown

A similar difference was also detected for the leaf extracts. Due to constraints in material, we just tested the same concentration of aqueous extract in bark, without trying to reach stronger effects by raising the concentration. Again, leaf extract from Sanankoroba (Fig. 5b) was more efficient over the extract from Sikasso (Fig. 5c).

**Fig. 6:**
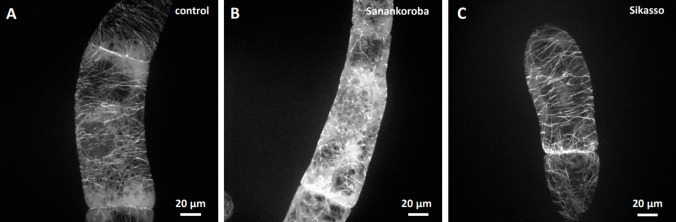
Effect of aqueous extracts (0.5 mg. mL^−1^) from roots of *D. microcarpum* collected at two different sites in Mali in GFP-TuA3. Treatment time was 2 h **a** mock control **b** extract from roots collected in Sanankoroba **c** extract from roots collected in Sikasso. Geometrical projections from confocal z-stacks are shown

For root extract, the difference was most noteworthy. Here, the extract from Sikasso (Fig. 6c) did not produce a significant difference from the negative control (Fig. 6a), whilst the extract from Sanankoroba perturbed microtubules considerable, accompanied by a conspicuous increase of soluble background fluorescence.

**Fig. 7:**
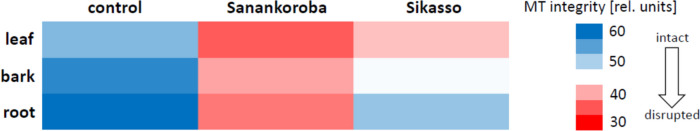
Quantification of the antimicrotubular effect of aqueous extracts (0.5 mg^.^mL^−1^) from different parts of *D. microcarpum* collected at the two sites in Mali. A score of 30 (red) corresponds to complete elimination, a score of 60 (blue) corresponds to fully intact microtubules. Treatment time was 2 h, target were tobacco BY-2 cells expressing a N-terminal fusion of GFP to tobacco tubulin α3 under control of the CaMV 35S promoter to visualise microtubules in vivo. Data represent mean values from 13–32 individual cells per experiment

In order to compare the differences in bioactivity depending on source tissue and collection site, we quantified microtubule integrity and plotted a heat map, whereby blue colours represent low activity (high integrity of microtubules), whilst red colours represent microtubule disruption. Irrespective of the tissue, the extracts obtained from plants collected at Sanankoroba were significantly more active than those from Sikasso (Fig. 7). With respect to the source tissue, the leaf extracts were most active, irrespective of sampling site, whilst bark and root extracts were less efficient. Here, the relative efficiency differed between the sampling sites. Whilst roots from plants collected at Sanankoroba were more active than bark extract, this relationship was inversed in case of plants collected at Sikasso.

### Genetic barcoding shows a very close phylogenetic relationship

**Fig. 8:**
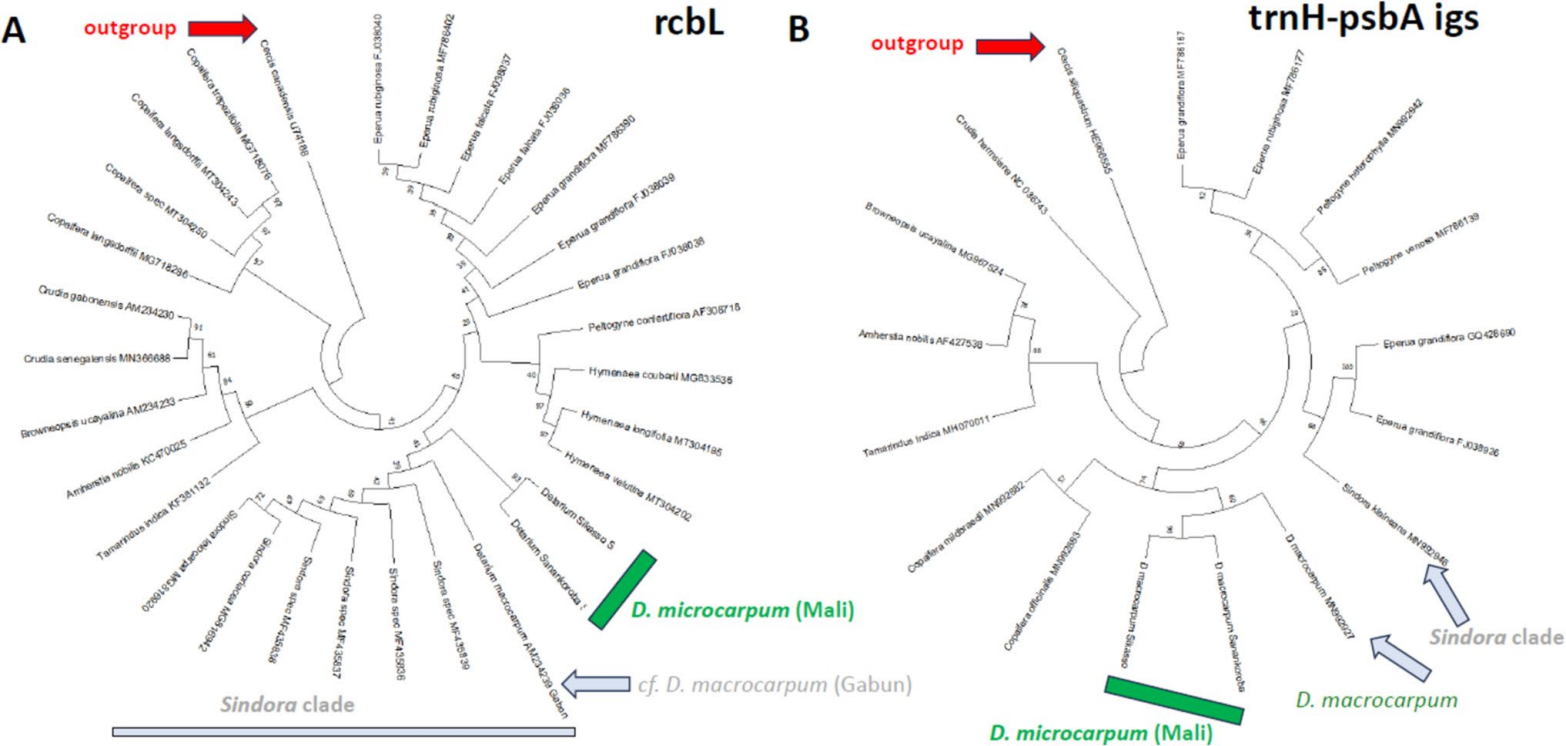
Phylogenetic position of the two accessions for *D. macrocarpum* collected in Mali (highlighted in green) compared to other members of the Tribus *Detarieae* and *Cercis canadensis* (Tribus Cercidoideae) as outgroup based on the plastidic marker *rbcL* (**a**) and *trnH-psbA igs* (**b**). The phylogeny was constructed using the Neighbour-Joining algorithm and bootstrap values derived from 1000 burn-ins. Grey: accession Breteler 12528 (WAG) from Gabun declared as *D. macrocarpum*, but partially clustering with the morphologically similar *Sindora klaineana*

We used two genetic barcodes, the large subunit of *ribulose-bis-phosphate carboxylase* (*rbcL*) and the spacer region between the t-RNA coding gene *trnH* and the photosystem-II component *psbA* (*trnH-psbA igs*), to infer the phylogenetic relationship between the two *D. microcarpum* accessions from Mali and their position with respect to other members of the Tribus Detariae (Fig. 8). For both markers, the two accessions clustered very closely into the same clade, and were clearly separated from *D. macrocarpum*, a closely related species from Gabon. For the rbcL marker (Fig. 8A), this *D. macrocarpum* accession was even clustering with *Sindora kleiniana*, the only West African member of the genus *Sindora*, which otherwise is found in South-East Asia. In case of the *trnH-psbA igs* marker, this accession was clustering with the two *D. microcarpum* accessions from Mali and was separated from the *Sindora* clade, which might be linked with the fact that this marker gives a higher resolution compared to the more conserved *rbcL* marker. Overall, the two accessions are very closely related and belong to the same species, which can be clearly delineated from *D. macrocarpum*. Although sequences for *D. senegalense*, the third species of the genus, were not available, it is very unlikely that the two accessions fall to that taxon. First, *D. senegalense* does not occur in arid regions such as Sanankoroba. Second, it differs significantly in morphology from *D. microcarpum*. Thus, the differences between the two accessions with respect to their anti-microtubular activity is very unlikely to be caused by a scenario, where the material collected in Sanankoroba and in Sikasso originate from different species of *Detarium*.

### The anti-microtubular effect involves the formation of tubulin speckles

**Fig. 9:**
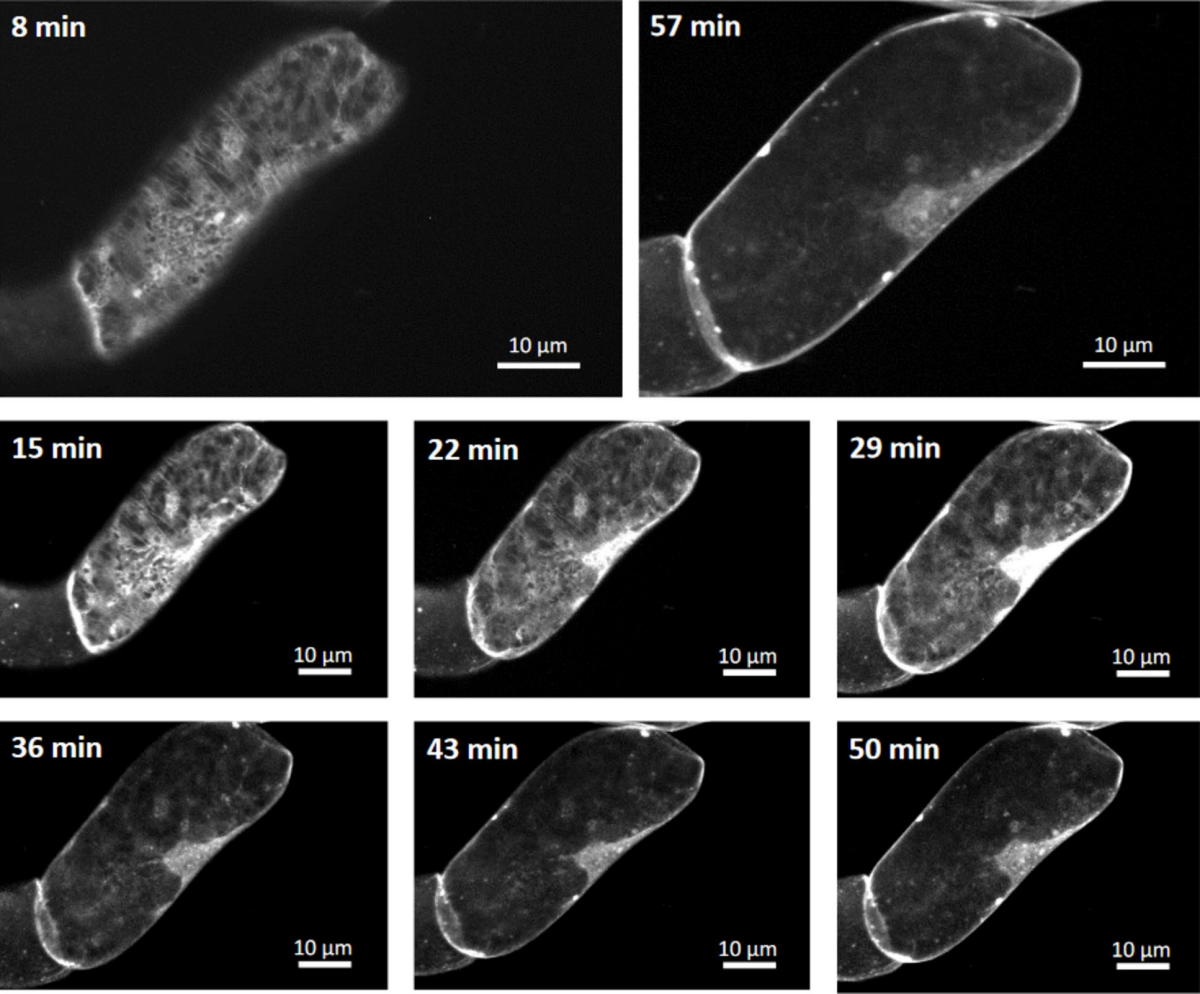
Time course for the antimicrotubular effect of aqueous extracts (5 mg. mL^−1^) from bark of *D.microcarpum* (collection site Sikasso) in tobacco BY-2 cells expressing a N-terminal fusion of GFP to tobacco tubulin α3 under control of the CaMV 35S promoter to visualise microtubules *in vivo*. Geometrical projection of z-stacks collected at a step size of 0.5 μm

To get insight into the mechanism of microtubule elimination, we conducted a time-course study, where we followed individual cells through the first hour following the application of the extracts. As to be able to detect potential transitional states material collected at Sikasso was used, because, here, the effect was milder. To get a clear time point zero, the cells were thoroughly mixed after addition of the compound, and then mounted on the observation slide. It required a few minutes to adjust the specimen for the time-lapse series, such that the first observation point was in the range of 5–10 min. Already at that point, microtubules appeared to be impaired, evident from very thin bundles, and the presence of diffuse fluorescence that was not organised in microtubules (Fig. 9). In the subsequent time interval (until around 30 min after application), the thinning of cortical microtubules continued, whilst a bright fluorescent signal accumulated around the nucleus. In addition, agglomeration of dark granules surrounded by a bright halo appeared in the cytoplasm. From 30 min onwards, cortical microtubules eclipsed completely, and also the fluorescence around the nucleus and the granules vanished. Instead, brightly labelled, but scarce, speckles appeared in the cortical cytoplasm and persisted even around 1 h after addition of the compound. This pattern clearly differed from the diffuse fluorescence seen after treatment with oryzalin (Fig. 2E, F). Over the course of the experiment, cells flattened, which became manifest in a larger area visible in the confocal section.

### The anti-microtubular effect is linked with alkaloids

**Fig. 10:**
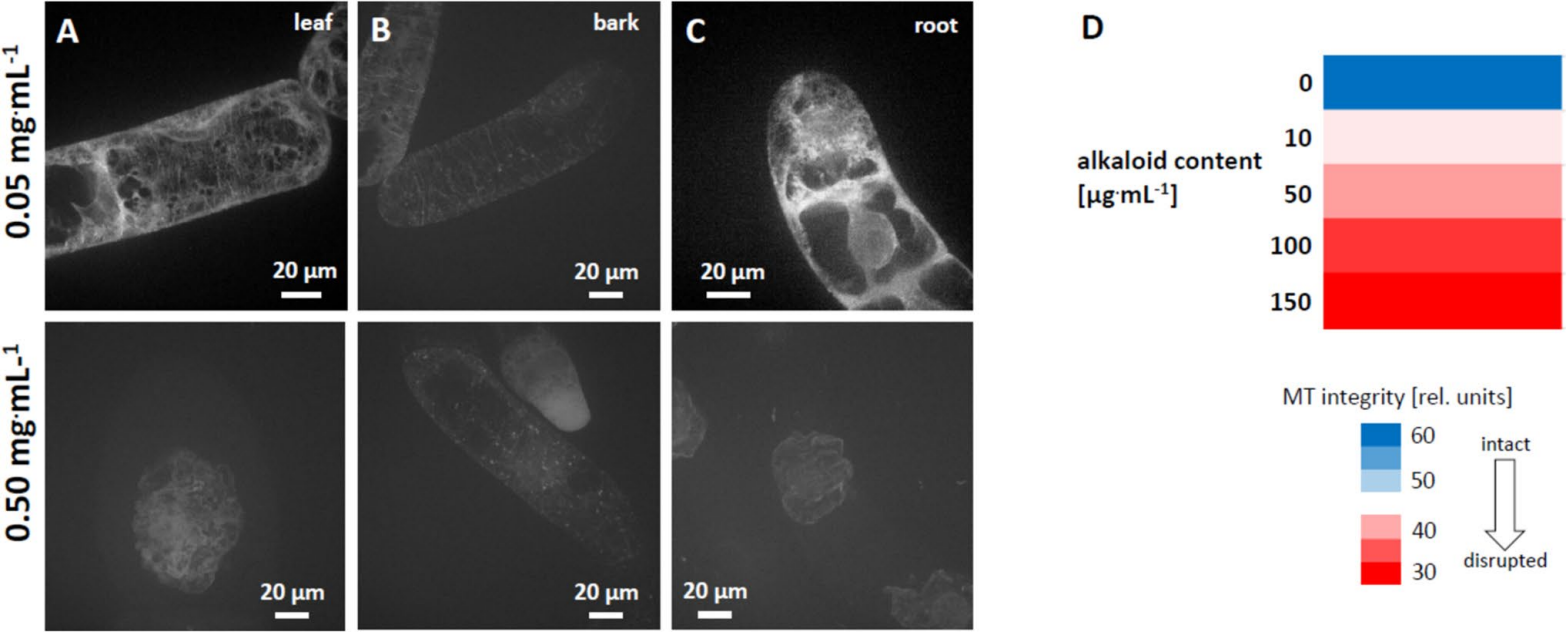
Antimicrotubular effect of the alkaloid fraction from different parts of *D. microcarpum* collected at Sanankoroba. **A**–**C** show representative cells after treatment with either 0.05 mg.mL^−1^ or with 0.50 mg^.^mL^−1^ of alkaloid fractions generated either from leaf (**A**), bark (**B**) or root (**C**). Treatment time was 2 h, target were tobacco BY-2 cells expressing a N-terminal fusion of GFP to tobacco tubulin □3 under control of the CaMV 35S promoter to visualise microtubules in vivo. **D** Dose–response relation of the antimicrotubular effect for alkaloids extracted from roots of *D. microcarpum* collected at Sanankoroba. A score of 30 (red) corresponds to complete elimination, a score of 60 (blue) corresponds to fully intact microtubules. Data represent mean values from 10 to 19 individual cells per experiment

As first step of activity-guided fractionation in order to identify the molecular base of the anti-microtubular effect, we tested the alkaloid fraction for activity (Fig. 10). In fact, we observed that alkaloid fractions from leaf, bark and root eliminated microtubules within 2 h at concentrations as low as 50 µg^.^mL^−1^ for bark alkaloids (Fig. 10B), whilst higher concentrations were required for leaf (Fig. 10A) and root (Fig. 10C) alkaloids. However, even those, less potent, alkaloid fractions, eliminated microtubules completely, when the concentration was raised to 500 µg^.^mL^−1^. Here, the fluorescent signal was decaying to such an extent that exposure time needed to be prolonged by an order of magnitude to catch an image of the cell, leading to a stronger background signal compared to the images collected for 50 µg^.^mL^−1^. For root alkaloids, the material was sufficient to conduct a dose–response study (Fig. 10D). Microtubule integrity was affected in a dose-dependent manner with a threshold at 10 µg^.^mL^−1^ and saturation from 100 µg^.^mL^−1^. If the effect of the alkaloid fraction is compared to the aqueous extract (Fig. 7), which was administered at 500 µg^.^mL^−1^, a concentration of 50 µg^.^mL^−1^ alkaloid fraction eliminated microtubules to a comparable degree. In other words, the alkaloid fraction is around one order of magnitude more potent than the aqueous extract, such that it is straightforward to assume that the active compound is residing in the alkaloid fraction.

### The rapid anti-microtubular effect of alkaloids is followed by long-term cytotoxicity

**Fig. 11:**
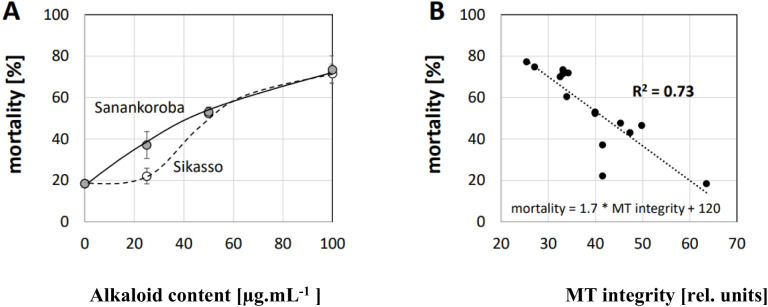
Cytotoxic effect of alkaloid fractions from different parts of *D. microcarpum* collected at Sikasso and Sanankoroba. **A** Dose–response relation of mortality in tobacco BY-2 cells expressing a N-terminal fusion of GFP to tobacco tubulin □3 under control of the CaMV 35S scored at day 3 after addition of the alkaloid fraction generated from leaves collected either in Sanankoroba or in Sikasso. Data represent mean and standard error from three independent experimental series. **B** Relationship between microtubule integrity scored at 2 h after addition of the alkaloid fraction and the mortality scored after day 3 induced by the same fraction pooled over fractions generated from leaves, bark and roots collected at the two sites. The negative correlation of mortality over MT integrity has been inferred by linear regression with a correlation coefficient *R*.^2^ = 0.73

The elimination of microtubules might be transient, or it might be irreversible. If it were transient, the effect on cellular physiology would be expected to be mild, because after some time new microtubule arrays would be formed. If the effect were irreversible, the impact would be expected to be more severe. Especially, the breakdown of mitotic spindles might cause a mitotic catastrophe initiating programmed cell death. For this reason, we assessed the mortality at day 3 after addition of the alkaloids (exploratory experiments had shown that the mortality during the first two days of the cultivation cycle was negligible). This time interval usually is sufficient to proceed through three complete cell cycles, such that mortality induced by a mitotic catastrophe should become detectable. A dose–response curve of mortality over the concentration of alkaloids (Fig. 11A) showed a dose-dependent increase of mortality to almost 80% for 100 µg^.^mL^−1^. As observed for the anti-microtubular effect (Fig. 10), the alkaloid extract generated from the samples collected in Sanankoroba were more potent compared to those from Sikasso, albeit at higher concentrations, the two curves converged. To assess, how mortality of a given alkaloid fraction related to its anti-microtubular effect, we plotted the mortality against the values for microtubule integrity (Fig. 11B). This plot revealed that the degree of microtubule disintegration at 2 h after addition of the alkaloid fraction correlated well with the cellular mortality observed 3 days later (*R*^2^ = 0.73). Thus, the anti-microtubular activity was a good predictor for the ensuing cytotoxicity.

## Discussion

In the current work, we could show that extracts from *D. microcarpum*, a medicinal plant in Mali traditionally used against cancer are able to eliminate microtubules in tobacco BY2 cells. This activity depends on source tissue and collection site and can be attributed to the alkaloid fraction. This leads to a couple of questions: What can we conclude on the mode of action for the bioactivity against microtubules? How does this bioactivity relate to the ethnomedicinal use of this and related plants? How can the results of this work be valorised for application?

### Microtubule nucleation as potential target?

If assessed after 2 h, microtubules are eliminated and tagged soluble tubulin is observed as green background in the cytoplasm. At first sight, the diffuse fluorescent background observed in parallel to the elimination of microtubules (Fig. 2) resembles the pattern produced by dinitroaniline herbicides, such as trifluralin and oryzalin. These compounds are known to sequester tubulin heterodimers from incorporation into the plus-end of the growing microtubules (Morejohn et al. 1987), such that the microtubules disappear based on the degree of their dynamic equilibrium. However, the fluorescent speckles, seen as transitional states during the time-course experiment (Fig. 9) are not supporting tubulin sequestering as mode of action. Such speckles, by their appearance and localisation, can be interpreted as tubulin nucleation sites. Whilst microtubules in animal cells are mainly (but not exclusively) nucleated at centrosomes, cells of higher plants lack centrosomes. However, the ring complex consisting of the deviant □-tubulin and five related proteins has been conserved in plant cells as well (for review see Lee and Liu 2019). The minimal nucleus consists of four □□-heterodimers at this so-called □-tubulin ring complex (for review see Sulimenko et al. 2022) but requires interaction with microtubule-associated proteins, such as MAP215 (Thawani et al. 2020) to achieve the transition into elongation. In fact, there is a plant homologue of MAP215, MOR1 (Chen et al. 2022). To test, whether the effect of *D. microcarpum* extract targets nucleation, it would be interesting to use GFP-tagged versions of End-Binding Protein 1 (Ouko et al. 2010), which would allow to see, whether the life-time of microtubules is altered prior to their elimination.

### How does this bioactivity relate to the ethnomedicinal use of this and related plants?

*D. microcarpum* is an essential component of traditional medicine in West Africa. As other traditional medicinal plants, the type of use is very diverse and depending on region and cultural context. In addition, the fruits are consumed as functional food, whilst other plant parts are used for healing. For Mali, where this tree is known as *N’tabacoumb*a, a recent ethnopharmaceutical study (Dembele et al. 2022) uncovered more than ten applications, mostly linked with infectious diseases, such as dysentery, bilharziosis or meningitis, but also applications pointing to hormone-like activities, such as difficult delivery, painful periods or female bellyaches. A traditional use of *D. microcarpum* against cancers is reported for Northeast Nigeria (Mohammed et al. 2014) under the name *Taura* (Hausa), *Gatafo* (Kanur) or *Gwalangwalan* (Bura). To what extent the use of this tree against cancers is a regional specificity of Nigeria, is difficult to judge, because the knowledge about traditional medicine is passed on within specific healing traditions that are secluded to the outer world (Atawodi et al. 2002). Since *D. microcarpum* seems to be used for traditional cancer therapy in Cameroon as well (Adjanohoun 1996), it is at least feasible to assume a larger therapeutic use of this tree against cancers.

A study in breast cancer cells demonstrated that bark extracts from *D. microcarpum* induce apoptotic cell death (Mohammed et al. 2016). This was preceded by activation of the SAPK/JNK phosphorylation cascade resulting in a hyper-phosphorylation of the MAP kinase p48 triggering the mitochondrial permeability pore and leakage of cytochrome C into the cytoplasm. In contrast to the antibacterial activity, were a specific labdane diterpenoid, named microcarpine and its ceramide derivative microcarpamide (Fouatio Feudjou et al. 2022) have been proposed as active compound, the molecule responsible for the apoptotic effect remains to be elucidated by activity-guided fractionation.

Nevertheless, the data from the current study allow to suggest a functional hypothesis on the mode of action. Three aspects are relevant here: (1) Microtubules are eliminated. (2) This elimination shows hallmarks of impaired microtubule nucleation. (3) The elimination of microtubules is followed by programmed cell death. These aspects point to a scenario, where the extracts of *D. microcarpum* can induce mitotic catastrophe.

Apoptosis in consequence of mitotic arrest is a common phenomenon in animal cells (reviewed in Castedo et al. 2004; Kastan and Bartek 2004), and underlies, for instance, the anti-tumour activity of paclitaxel (Jordan et al. 1996). A breakdown of spindle polarity seems to be crucial, since cell death is often preceded by the occurrence of multipolar spindles (Roninson et al. 2001), and since cancer cells often circumvent this by centriole clustering. In a previous study, we identified gallic acid as anti-microtubular compound from extracts of wild Citrus species which is used in Traditional Chinese Medicine (TCM) for tumour therapy by an activity-guided fractionation strategy (Tan et al. 2015). Gallic acid was able to suppress centriole clustering in HeLa cells, leading to tripolar spindles and subsequent apoptotic death, whilst fibroblast cells that are endowed with a primary bipolar spindle, remained unaffected. A similar approach led to the discovery of glucobrassinolide as compound responsible for the anti-microtubule activity of *Isatis tinctoria*, a plant used in TCM to cure viral diseases including Covid, possibly by interfering with microtubule-dependent transport of viral RNA (Guan et al. 2021). This compound as well is able to induce the apoptosis of HeLa cells.

Since the cells of higher plants lack centrioles, it has been questioned, whether programmed cell death in consequence of mitotic arrest exists. There is progressive evidence that this is the case. For instance, cadmium can induce programmed cell death in tobacco BY-2 cells depending on the phase of the cell cycle (Kuthanová et al. 2008). If administered at the G_2_-M transition, the cells display hallmarks of programmed cell death, such as DNA laddering. Likewise, the phytohormone kinetin can induce the elimination of cortical microtubules in tobacco BY-2 cells, which is then followed by programmed cell death (Kaźmierczak et al. 2023). Our finding that the alkaloid extracts from *D. microcarpum* can induce, in the same cell strain, a mortality in tight correlation with their effect on microtubule integrity (Fig. 11) adds a further facet to the occurrence of mitotic catastrophe in plant cells. It is possible to follow the loss of membrane integrity in the plasma membrane in parallel to the breakdown of nuclear envelope integrity using a dual visualisation with Acridin Orange and Ethidium Bromide (Sarheed et al. 2020), which would allow further insight into the cellular sequence of events. Furthermore, once molecular candidates for the active compounds have been identified, one could use cycling tobacco cells to detect effects on mitotic index, but also malformations of the spindle similar to a previous study on glucobrassicine, a bioactive compound identified in a TCM plant (Guan et al. 2021).

### Towards evidence-based traditional medicine for developing countries

The active compound underlying the anti-microtubular effect of *D. microcarpum* has not been identified yet. The availability of the fluorescently tagged microtubule marker line and the swift readout enables a strategy based on bio-activity guided fractionation. This strategy has been employed successfully to identify glucobrassicine as active compound mediating the anti-microtubular effect of Northern Ban Lan (Isatis tinctoria), a plant used in Traditional Chinese Medicine to treat viral infections of the respiratory tract (Guan et al. 2021). Based on our finding, the alkaloid fraction of *D. microcarpum* would provide a good starting point for this type of approach. Despite the huge potential of plant secondary products, phytotherapy is often excluded from the official medicinal canon in the industrialised countries, because active compounds, mode of action and clinical studies on efficacy and safety are often unknown (for review see Fürst and Zündorf 2015). On the other hand, the rigorous regulations effective in industrialised countries often do not make sense under the conditions of developing countries, where public health insurance systems are often missing and where large parts of the population do not have access to evidence-based therapy. The World Health Organization has addressed this gap in a series of conferences and also guidelines targeted to reconcile quality and safety requirements with traditional phytotherapy (reviewed in Ameh et al. 2010). A first step is the definition of the local medicinal plants by clear descriptions of their morphology, anatomy, and, if known, chemical composition in form of pharmacopeias. Functional assays (such as the microtubule integrity test developed in the current work), combined with information on active compounds (to be identified by activity-guided fractionation studies) could help to define standards that can be verified by quality control. Whilst this might still not suffice to meet the regulatory requirements for therapeutic use in the medicinal system of industrialised countries, it would represent a decisive step forward to secure therapeutic quality in developing countries. Initiatives as the MTA (Médicaments Traditionnels Améliorés) developed by the Department of Traditional Medicine in Bamako, Mali (Sanogo et al. 2023) represent initiatives into that direction that can be developed and extended by integrating chemical and mechanistic knowledge about these plants and their usage, but also by integrating genetic barcoding as strategy to authenticate the plant material and safeguard it against surrogation and adulteration.

## References

[CR1] Adebayo IA, Gagman HA, Balogun WG, Adam MAA, Abas R, Hakeem KR, Nik Him NAIIB, Samian MRB, Arsad H (2019) *Detarium microcarpum*, *Guiera senegalensis*, and *Cassia siamea* induce apoptosis and cell cycle arrest and inhibit metastasis on MCF7 breast cancer cells. Evid Based Complement Alternat Med 2019:6104574 31239861 10.1155/2019/6104574 PMC6556270

[CR2] Adjanohoun E (1996) Traditional medicine and pharmacopoeia: contribution to ethnobotanical and floristic studies in Cameroon. Scientific, Technical and Research Commission of the Organization of African Unity, Lagos Nigeria, pp 301–325

[CR3] Altmann KH, Gertsch J (2007) Anticancer drugs from nature—natural products as a unique source of new microtubule-stabilizing agents. Nat Prod Rep 24:327–357 17390000 10.1039/b515619j

[CR4] Ameh SJ, Obodozie OO, Inyang US, Abubakar MS, Garba M (2010) Current phytotherapy - a perspective on the science and regulation of herbal medicine. J Med Plant Res 4:72–81

[CR5] Atawodi SE, Ameh DA, Ibrahim S, Andrew JN, Nzelibe HC, Onyike EO, Anigo KM, Abu EA, James DB, Njoku GC, Sallau AB (2002) Indigenous knowledge system for treatment of trypanosomiasis in Kaduna state of Nigeria. J Ethnopharmacol 79:279–282 11801393 10.1016/s0378-8741(01)00351-8

[CR6] Bernardini S, Tiezzi A, Laghezza Masci V, Ovidi E (2018) Natural products for human health: an historical overview of the drug discovery approaches. Nat Prod Res 32:1926–1950 28748726 10.1080/14786419.2017.1356838

[CR7] Castedo M, Perfettini JL, Roumier T, Andreau K, Medema R, Kroemer G (2004) Cell death by mitotic catastrophe: a molecular definition. Oncogene 23:2825–2837 15077146 10.1038/sj.onc.1207528

[CR8] CBOL Plant Working Group (2009) A DNA barcode for land plants. Proc Natl Acad Sci USA 106:12794–12797 19666622 10.1073/pnas.0905845106 PMC2722355

[CR9] Chen Y, Liu X, Zhang W, Li J, Liu H, Yang L, Lei P, Zhang H, Yu F (2022) MOR1/MAP215 acts synergistically with katanin to control cell division and anisotropic cell elongation in Arabidopsis. Plant Cell 34:3006–3027 35579372 10.1093/plcell/koac147 PMC9373954

[CR10] Dembele N (2023) Etudes Ethnobotanique, Phytochimique Et Activités Biologiques De Detarium Microcarpum Guill & Perr (Fabaceae) Et Flueggea Virosa (Roxb. Ex Willd.) Voigt (Euphorbiaceae), Deux Plantes Medicinales Recoltees Au Mali.” Bamako: Université Des Sciences, Des Techniques Et Des Technologies De Bamako

[CR11] Dembele N, Somboro A, Badiaga M, Cisse S, Cisse M, Togola I, Nick P, Traore N (2022) Ethnobotanical survey and phytochemical screening of aqueous extracts from *Detarium microcarpum* Guill. Perr in Mali. J Medicin Plants Res 16:280–287

[CR12] Dogara AM (2022) Biological activity and chemical composition of Detarium microcarpum Guill. and Perr – a systematic review. Adv Pharmacol Pharmaceut Sci 2022:7219401. 10.1155/2022/7219401 10.1155/2022/7219401 PMC9569227 36254172

[CR13] Doyle JJ, Doyle JL (1987) A rapid DNA isolation procedure for small quantities of fresh leaf tissue. Phytochem Bull 19:11–15

[CR14] Fonrose X, Ausseil F, Soleilhac E, Masson V, Davin B, Pouny I, Cintrat JC, Rousseau B, Barette C, Massiot G, Lafanechére L (2007) Parthenolide inhibits tubulin carboxypeptidase activity. Cancer Res 67:3371–3378 17409447 10.1158/0008-5472.CAN-06-3732

[CR15] Fouatio Feudjou W, Mbock AM, Tedjon Sielinou V, Fouotsa H, Njonté Wouamba SC, Kamkumo Gounoue R, Freeze M, Stammler HG, Kezeutas Bankeu JJ, Pierre M, Ndjakou Lenta B, Tiabou Tchinda A, Sewald N, Nkengfack AE (2022) Secondary metabolites from *Detarium microcarpum* Guill. and Perr. (Fabaceae). Z Naturforsch C 77:253–261 35212491 10.1515/znc-2021-0239

[CR16] Fürst R, Zündorf I (2015) Evidence-based phytotherapy in europe: where do we stand? Plant Med 81:962–967 10.1055/s-0035-1545948 25922913

[CR17] Gaff D, Okong’o-Ogola O (1971) The use of non-permeating pigments for testing the survival of cells. J Exp Bot 22:756–758

[CR18] Goabaone G, Venkataraman S, Brown PD, Masisi K, Kwape TE, O’Nkwe D, Rantong G, Makhzoum A (2023) The use of African medicinal plants in cancer management. Front Pharmacol 14:1122388 36865913 10.3389/fphar.2023.1122388 PMC9971233

[CR19] Guan P, Zhou J, Girel S, Zhu X, Schwab M, Zhang K, Wang-Müller QY, Bigler L, Nick P (2021) Anti-microtubular activity of the TCM herb Northern Ban Lan (*Isatis tinctoria*) leads to Glucobrassicin. J Int Plant Biol 63:2058–2074 10.1111/jipb.13177 34636476

[CR20] Guedje NM, Tadjouteu F, Dongmo RF (2013) Medecine traditionelle africaine (MTR) et phytomedicaments: défis et strategies de developpement. Health Sci Disease 13. https://www.hsd-fmsb.org/index.php/hsd/article/view/99. Accessed 23 Nov 2023

[CR21] Gurib-Fakim A (2006) Medicinal plants: traditions of yesterday and drugs of tomorrow. Mol Asp Med 27:1–93 10.1016/j.mam.2005.07.008 16105678

[CR22] Himmelspach R, Wymer CL, Lloyd CW, Nick P (1999) Gravity-induced reorientation of cortical microtubules observed *in vivo*. Plant J 18:449–453 11536906 10.1046/j.1365-313x.1999.00467.x

[CR23] Jordan MA, Wendell K, Gardiner S, Derry WB, Copp H, Wilson L (1996) Mitotic block induced in HeLa cells by low concentrations of paclitaxel (Taxol) results in abnormal mitotic exit and apoptotic cell death. Cancer Res 56:816–825 8631019

[CR24] Kastan MB, Bartek J (2004) Cell-cycle checkpoints and cancer. Nature 432:316–323 15549093 10.1038/nature03097

[CR25] Kaźmierczak A, Siatkowska E, Li R, Bothe S, Nick P (2023) Kinetin induces microtubular breakdown, cell cycle arrest and programmed cell death in tobacco BY-2 cells. Protoplasma 260:349–369 36239807 10.1007/s00709-022-01814-6 PMC10125952

[CR26] Klein-Júnior LC, van der Heyden Y, Henriques AT (2016) Enlarging the bottleneck in the analysis of alkaloids: a review on sample preparation in herbal matrices. TrAC Trends Anal Chem 80:66–82

[CR27] Kumagai F, Yoneda A, Tomida T, Sano T, Nagata T, Hasezawa S (2001) Fate of nascent microtubules organized at the M/G_1_ interphase, as visualized by synchronized tobacco BY-2 cells stably expressing GFP-tubulin: time-sequence observations of the reorganization of cortical microtubules in living plant cells. Plant Cell Physiol 42:723–732 11479379 10.1093/pcp/pce091

[CR28] Kuthanová A, Fischer L, Nick P, Opatrný Z (2008) Cell cycle phase specific death response of tobacco BY-2 cell line to cadmium treatment. Plant Cell Environ 31:1634–1643 18721263 10.1111/j.1365-3040.2008.01876.x

[CR29] Lee YJ, Liu B (2019) Microtubule nucleation for the assembly of acentrosomal microtubule arrays in plant cells. New Phytol 222:1705–1718 30681146 10.1111/nph.15705

[CR30] Mizuno K, Suzuki T (1991) Effects of anti-microtubule drugs on in-vitro polymerization of tubulin from mung bean. Bot Mag Tokyo 103:435–448

[CR31] Mohammed ZK, Daja M, Hamza HG, Gidado A, Hussaini IM (2014) Ethnomedicinal survey of folkloric plants used in managing breast cancers by the traditional medical practitioners of North-East Nigeria. J Med Appl Bioscience 6:29–43

[CR32] Mohammed ZK, Templeton D, Hamza HG, Gidado A, Hussaini IM (2016) *Detarium microcarpum* stem-bark extracts induce apoptosis in human breast adenocarcinoma MDA-MB 231 cells via cJNK activation and mitochondrial cytochrome C release. J Pharm Biomed Sci 6:439–444

[CR33] Mondal A, Gandhi A, Fimognari C, Atanasov AG, Bishayee A (2019) Alkaloids for cancer prevention and therapy: current progress and future perspectives. Eur J Pharmacol 858:172472 31228447 10.1016/j.ejphar.2019.172472

[CR34] Morejohn LC, Bureau TE, Molè-Bajer J, Fosket DE (1987) Oryzalin, a dinitroaniline herbicide, binds to plant tubulin and inhibits microtubule polymerization in vitro. Planta 172:252–264 24225878 10.1007/BF00394595

[CR35] Newman DJ, Cragg GM, Snader KM (2003) Natural products as sources of new drugs over the period 1981–2002. J Nat Prod 66:1022–1037 12880330 10.1021/np030096l

[CR36] Nick P (2008) Control of Cell Axis. Plant Cell Monogr 143:3–46

[CR37] Nick P, Heuing A, Ehmann B (2000) Plant chaperonins: a role in microtubule-dependent wall-formation? Protoplasma 211:234–244

[CR38] Ouko MO, Sambade A, Brandner K, Ahad A, Heinlein M, Nick P (2010) Tobacco mutants with reduced microtubule dynamics are less susceptible to TMV. Plant J 62:829–839 20230489 10.1111/j.1365-313X.2010.04195.x

[CR39] Owellen RJ, Hartke CA, Dickerson RM, Hains FO (1976) Inhibition of tubulin-microtubule polymerization by drugs of the Vinca alkaloid class. Cancer Res 36:1499–1502 1260766

[CR40] Page JE, Balza F, Nishida T, Towers GN (1992) Biologically active diterpenes from *Aspilia mossambicensis*, a chimpanzee medicinal plant. Phytochemistry 10:3437–3439 10.1016/0031-9422(92)83702-z 1368857

[CR41] Parness J, Horwitz SB (1981) Taxol binds to polymerized tubulin *in vitro*. J Cell Biol 91:479–487 6118377 10.1083/jcb.91.2.479 PMC2111958

[CR42] Roninson IB, Broude EV, Chang B-D (2001) If not apoptosis, then what? Treatment-induced senescence and mitotic catastrophe in tumor cells. Drug Resist Updat 4:303–313 11991684 10.1054/drup.2001.0213

[CR43] Sanogo R, Dembele D, Doumbia S, Mariko ABA, Fofana MY (2023) Médicaments Traditionnels Améliorés (MTA) sous forme de pommade au Mali. J Afric Technol Pharmaceut Biopharm 2:22–23

[CR44] Sarheed MM, Rajabi F, Kunert M, Boland W, Wetters S, Miadowitz K, Kaźmierczak A, Sahi VP, Nick P (2020) Cellular base of mint allelopathy: menthone affects plant microtubules. Front Plant Sci 11:546345 33042176 10.3389/fpls.2020.546345 PMC7524878

[CR45] Shen B (2015) A new golden age of natural products drug discovery. Cell 163:1297–1300 26638061 10.1016/j.cell.2015.11.031 PMC5070666

[CR46] Sulimenko V, Dráberová E, Dráber P (2022) γ-Tubulin in microtubule nucleation and beyond. Front Cell Dev Biol 10:880761 36158181 10.3389/fcell.2022.880761 PMC9503634

[CR47] Tan S, Grün C, Guan X, Zhou Z, Schepers U, Nick P (2015) Gallic acid induces mitotic catastrophe and inhibits centrosomal clusteringin HeLa cells. J Toxicol in Vitro 30:506–513 10.1016/j.tiv.2015.09.011 26368671

[CR48] Thawani A, Rale MJ, Coudray N, Bhabha G, Stone HA, Shaevitz JW, Petry S (2020) The transition state and regulation of γ-TuRC-mediated microtubule nucleation revealed by single molecule microscopy. Elife 9:e54253 32538784 10.7554/eLife.54253 PMC7338055

[CR49] World Health Organization (WHO). Global health estimates 2020: deaths by cause, age, sex, by country and by region, 2000–2019. WHO; 2020. https://www.who.int/fr/news-room/fact-sheets/detail/cancer. Accessed 11 Sept 2023

